# Dehydromiltirone inhibits osteoclast differentiation in RAW264.7 and bone marrow macrophages by modulating MAPK and NF-κB activity

**DOI:** 10.3389/fphar.2022.1015693

**Published:** 2022-09-21

**Authors:** Wei Deng, YanBo Huang, HaiShang Li, ChiWei Chen, YueWei Lin, Min Wang, HuaSheng Huang, Teng Liu, QiuLi Qin, Yang Shao, YongChao Tang, Kai Yuan, JinYong Ding, LiangLiang Xu, YongXian Li, ShunCong Zhang

**Affiliations:** ^1^ The First Clinical Academy, Guangzhou University of Chinese Medicine, Guangzhou, China; ^2^ The First Affiliated Hospital of Guangzhou University of Chinese Medicine, Guangzhou, China; ^3^ Lingnan Medical Research Center of Guangzhou University of Chinese Medicine, Guangzhou, China; ^4^ Guangdong Provincial Hospital of Chinese Medicine, The Second Affiliated Hospital of Guangzhou University of Chinese Medicine, Guangzhou, China

**Keywords:** dehydromiltirone, osteoclastogenesis, BMMS, MAPK, NF-κB, network pharmacology

## Abstract

**Background:** Osteoporosis is a type of systematic metabolic bone disease caused by the decrease in osteogenic activity or excessive resorption of bone with the relative enhancement of osteoclast function. As osteoporosis seriously affects the quality of patients’ life, effective drugs are needed to treat this disease. Based on the combination of network pharmacology and cellular studies, this study aimed to investigate the probable mechanism of Dehydromiltirone (DHT) in the treatment of osteoporosis.

**Method:** The targets of DHT in osteoporosis were searched using the PharmGKB, OMIM, and Genecard platforms. The PPI core targets, and the GO and KEGG enrichment analysis results were obtained using Cytoscape software, and the David and Metascape databases, respectively. The network pharmacology results were also verified *via in vitro* cellular experiments.

**Results:** Through network pharmacology and docking analysis, we found DHT was involved in peptide tyrosine phosphorylation, cell surface receptor tyrosine kinase signaling pathways, and MAPK signaling pathways. According to the molecular docking results, the binding of DHT to MAPK14 was more stable than other proteins, which suggests that DHT may affect osteoclast formation through the MAPK signaling pathway. Moreover, DHT was found to inhibit the expression of osteoclast-associated genes, including NFATc1, CTSK, c-Fos, Acp5, and MMP9; as well as the phosphorylation of P38, ERK, and JNK of the MAPK signaling pathway; and the degradation of IκB-α of NF-κB signaling pathway.

**Conclusion:** DHT exhibited an anti-osteoclastogenesis effect by reducing the expression of related genes, ultimately inhibiting bone resorption *in vitro*.

## 1 Introduction

Bone is a volatile equilibrium organ that constantly regenerates and resorbs. The basic condition for maintaining bone morphology and function is a harmonious balance between bone formation and absorption (Baron and Kneissel, 2013; Siddiqui and Partridge, 2016; Leong, 2018).

Osteoclasts are special monocyte macrophages that possess a bone resorption function. When the balance between osteogenesis and osteolysis is disrupted, numerous bone diseases occur, such as bone cancer, periodontal disease, and bone rarefaction (Rath et al., 2013; Lou et al., 2019; Li et al., 2021). In particular, when osteoclasts exert an excessive effect on bone resorption, osteoporosis is prone to occur and is distinguished by bone loss, destruction of the bone structure, and fragile fractures, ultimately leading to a burden on society and families (Rachner et al., 2011; Boudin and Van Hul, 2017; Bernard, 2019; Compston et al., 2019).

Owing to the development of bioinformatics, the concepts and models of network pharmacology have been further extended, transforming the traditional “Singlemode (target + drug)” into a “Multi-component (target + drug)” mode. This model uses modern information technology to develop the interaction mechanism, target prediction, and toxicology of traditional Chinese medicine (TCM) (Wu et al., 2016; Zhang et al., 2019). Therefore, we employed network pharmacology in this research predicting the drug-target interactions of Dehydromiltirone (DHT) and providing a theoretical basis for further experimental validation.

The traditional Chinese medicine*s, Salvia miltiorrhiza Bge* and *Salvia przewalskii Maxim* have been widely used to treat osteoporosis and other related diseases in the clinic. Other active ingredients have also been demonstrated to inhibit osteoporosis by blocking osteoclast production or promoting osteoblast growth (Guo et al., 2014; He et al., 2019; Ekeuku et al., 2021; Qin et al., 2021; Qu et al., 2021). DHT, a diterpenoid quinone found in the traditional Chinese medicines, *Salvia miltiorrhiza Bge* and *Salvia przewalskii Maxim*, has been widely used to prevent liver injury by modifying the MAPK and NF-κB signaling pathways, reducing neuroinflammatory responses, and inhibiting platelet aggregation. (Lin et al., 1988; Chen et al., 2003; Yang et al., 2011; Yue et al., 2014). Therefore, the mechanism by which DHT inhibits osteoclast formation is related to the MAPK pathway. The NF-κB pathway acts as a downstream pathway of MAPK and a classical pathway of osteoclasts, which are affected by the MAPK pathways that inhibit osteoclast production.

Owing to the vital role of osteoclasts in osteoporosis, and the anti-inflammatory effect of DHT, previous network pharmacology results and cellular and molecular experiments were employed to explore the mechanism of action of DHT in the prevention and treatment of osteoporosis.

## 2 Materials and methods

### 2.1 Network pharmacology data sources

#### 2.1.1 Structural formula of dehydromiltirone and prediction of related targets

According to the chemical name, “Dehydromiltirone (DHT),” and the CAS number “116064-77-8,” the target information was obtained by searching the TCMSP database (https://tcmsp-e.com/). The structural formula of DHT was also searched in the PubChem Data Bank (https://pubchem.ncbi.nlm.nih.gov/), saved in “PDF” format, and input into the SwissTargetPrediction database to obtain potential drug targets.

#### 2.1.2 Excavate OP-related targets

Using “Osteoporosis” as the keyword, relevant disease targets were obtained by searching PharmGKB (https://www.pharmgkb.org/), OMIM (https://omim.org/), and Genecards (https://www.genecards.org/). After deduplication and sorting, the data were imported into Excel tables and retained for future use. The potential targets and disease-related targets of DHT were then imported into the online website, Draw Venn Diagram (https://www.researchgate.net/), along with two intersected maps to obtain drug-disease common targets and generate Venn diagrams.

#### 2.1.3 Protein-protein interaction analysis

The common targets obtained in Section 2.1.2 were analyzed using the String database. The biological category was set to “*Homo Sapiens*,” the minimum interaction scores were set to 0.700, the free nodes were hidden, and the remaining parameters were kept as the default parameters of the system.

#### 2.1.4 Screening of the core targets

Using the CytoNCA plug-in in Cytoscape software (v.3.5.1), topological analysis was performed based on the degree centrality parameters. Local average connectivity-based method centralities, betweenness centrality, closeness centralities, network centralities, and core targets were obtained.

#### 2.1.5 GO enrichment and KEGG analysis

GO and KEGG enrichment analyses of the common targets were performed using the David and Metascape databases, respectively. The results of these analyses were uploaded to the Biological Information Data Analysis Platform, and histograms and bubble charts were generated for data visualization. The number of genes in the enriched pathway determines the size of the bubbles and the height of the columns in the plot. Colors represent *p*-values; the higher the enrichment, the smaller the *p*-value.

#### 2.1.6 Molecular docking between the core targets and naringin

DHT was selected as the ligand, and its small molecule two-dimensional structure in mol2 format was downloaded from the TCMSP website. The core gene in 1.4 was selected as the receptor, and the corresponding protein structure in PDB format was downloaded from the RCSB PDB database. AutoDock software (v.4.2) was used to make the molecular docking. The binding energy was used to assess the docking effect of the key target and active ingredients. In addition, the greater the binding energy, the better the binding activity of the ligand and receptor protein, and the more stable the docking state.

### 2.2 Osteoclast cultivation and experimental verification

#### 2.2.1 Materials and reagents

DHT (98% or higher purity) was extracted by ChemFaces (Wuhan, China). The DHT monomer was solubilized in dimethyl sulfoxide (DMSO, Sigma) to a concentration of 100 mM, stored at −80°C, and diluted with phosphate-buffered saline (PBS) buffer. Alpha Minimum Essential Medium (α-MEM), 100 U/ml penicillin, 100 U/ml/streptomycins (P/S), and fetal bovine serum (FBS, Cat # 10099141C) were acquired from Gibco (Carlsbad, CA). The Cell Counting Kit-8 (CCK-8, Cat # GK10001) was obtained from Glpbio (Montclair, CA). NFATc1(Cat # DF6446), c-Fos (Cat # AF5354), CTSK (Cat # DF6614), β-actin (Cat# AF7018), ERK (Cat# AF0155) Phospho-ERK1/2 (Thr202/Tyr204) (p-ERK,Cat# AF1055), JNK1/2/3 (Cat# AF6318) Phospho-JNK1/2/3 (Thr183 + Tyr185) (p-JNK,Cat# AF3318), P38 (Cat# AF6456), Phospho-p38 MAPK (Thr180/Tyr182) (p-P38, Cat# AF4001), IκB-α (Cat# AF5002), RANK (Cat # DF12532), and goat anti-rabbit IgG (H + L) HRP (Cat# S0001) were obtained from Affinity Biosciences Ltd. (Jiang Su, China). The Receptor Activator of Nuclear Factor-κ B Ligand (RANKL, Cat #315-11C) and Macrophage-Stimulating Factor (M-CSF, Cat # 500-P62G) were purchased from PeproTech (Rocky Hill, NJ). DAPI staining solution (Cat #C1005) was purchased from Beyotime Biotechnology Co., Ltd. (Shanghai, China). Fibrous actin (F-actin) was purchased from Beijing Bailui Polar Biotechnology Co. Ltd. (Cat # BN10063, Beijing, China). The tartrate-resistant acid phosphatase (TRAcP) stain Kit (Cat#G1492) was purchased from Solaibao Technology Co., Ltd. (Beijing, China). The EVO M-MLV Premix Kit (Cat #AG11706) and SYBR Premix (Cat #AG11721) were obtained from Accurate Biology (Hu Nan, China). The RAW 264.7 cell lineage (The Fifth Passage, Cat# JNO-3841) was obtained from Guangzhou Jennio Biotech Co., Ltd. (Guangzhou, China).

#### 2.2.2 *In vitro* osteoclastogenesis assay

The fifth-generation RAW264.7 cells were incubated in a complete medium containing 1% penicillin/streptomycins (P/S) and 10% FBS at 37°C in 5% CO_2_. When RAW264.7 cells reached 90%–95% confluence, they were subcultured.

Fresh bone marrow-derived macrophages (BMMs) were extracted from the upper and lower limbs of 4-week-old C57BL/6J mice according to the Guangzhou University of Chinese Medicine Animal Ethics Committee Guidelines (No. 20220527001), filtered, and then centrifuged with a 70 μm cell filter. A whole medium comprising M-CSF (50 ng/ml), 100 U/ml P/S, and 10% FBS was cultured for 6–7 days. Medium change was performed every other day.

#### 2.2.3 Cytotoxicity assays

RAW264.7 and BMMs were seeded in 96-well plates at 1 × 10^4^/well. After 48 h of treatment with different concentrations of DHT (0, 2.5, 5, 7, and 10 μM for RAW264.7; and 0, 0.625, 1.25, 2.5, and 5 μM for BMMs), 10 μl of CCK-8 was dispensed in each well according to the Cell Counting Kit-8 (CCK-8) instructions and incubated for 1 h without the light. The absorption of the sample was measured immediately at 450 nm by using a microplate reader. Thereafter, a histogram was generated, and the cytotoxicity and proliferation of DHT were evaluated using the absorbance values.

#### 2.2.4 TRAcP staining

RAW264.7 cells and BMMs were seeded in 96-well plates at 5 × 10^3^ cells/well. After 24 h, osteoclasts were cultured in complete α-MEM containing RANKL (50 ng/ml) and administered various concentrations of DHT for 6–7 days.

Media change was performed every 2 days and the osteoclasts were scoured three times with PBS until the formation of mature osteoclasts. Thereafter, the cells were fixed with 4% paraformaldehyde (Macklin, Shanghai, China) for 3–5 min, stained according to the TRAcP kit procedure, and photographed using a standard inverted microscope (Olympus, Japan). Mature osteoclasts were observed when more than three nuclei were present in the stained multinucleated cells.

#### 2.2.5 Bone resorption preparation

To ensure asepsis, beef bones are purchased at the market, cleaned, and placed in alcohol. Rinse the material overnight with complete medium containing the 1% P/S, and 10% FBS then place it in a 37-degree incubator to keep it dry. BMMs were induced for osteoclast differentiation in media containing RANKL (50 ng/ml) and M-CSF (50 ng/ml), and BMMs were transferred into 96-well plates containing bovine bone slices 3 days later.

On the second day, different concentrations of DHT (0,5,10 M) were used to intervene in the osteoclasts. After the osteoclasts had formed for 48 h, they were fixed with the electron microscope’s fixative solution and observed under the electron microscope (JEOL, ARM200F, JAPAN).

#### 2.2.6 Reverse transcription PCR

RAW 264.7 cells were seeded in 6-well plates at a consistency of 1 × 105/well and cultured with DHT (5 μM and 10 μM) and RANKL (50 ng/ml) for 6–7 days, with medium change performed every 2 days until the formation of mature osteoclasts. Total RNA was extracted by TRIZOL (Thermo Fisher Scientific, China) and the obtained RNA was reverse transcribed into cDNA by EVOM-MLV Premix Kit. PCR was performed using SYBR Green Kit and the following cycling conditions: 95°C, 30 min; 95°C, 5 s; 60°C, 30 min; and 50 cycles. All primers are listed in Table 1. All relevant gene expression results were processed using the RT-PCR instrument (Bio-Rad, United States). The 2^−ΔΔCT^ method was used to calculate the comparative expression levels of each gene.

**TABLE 1:** Primers for RT-PCR.

Gene	Forward (5-3)	Reverse (5-3)	Tm (m3)
NFATc1	GGA​GAG​TCC​GAG​AAT​CGA​GAT	TTG​CAG​CTA​GGA​AGT​ACG​TCT	60
c-Fos	GCGAGCAACTGAGAAGAC	TTGAAACCCGAGAACATC	60
CTSK	GGGAGAAAAACCTGAAGC	ATTCTGGGGACTCAGAGC	60
Acp5	TGTGGCCATCTTTATGCT	GTCATTTCTTTGGGGCTT	61
MMP9	CGT​GTC​TGG​AGA​TTC​GAC​TTG​A	TTG​GAA​ACT​CAC​ACG​CCA​GA	60
MMP13	TGT​TGC​TGC​CCA​TGA​GCT​TG	GGC​TTT​TGC​CAG​TGT​AGG​TA	61
β-actin	GGC​TGT​ATT​CCC​CTC​CAT​CG	CCA​GTT​GGT​AAC​AAT​GCC​ATG​T	61

#### 2.2.7 Western blot

RAW264.7 cells were cultivated in a 6-well board at a cell consistency of 1 × 10^6^ cells per well. On days 5, 3, and 1 post-seeding, the cells were cultured with DHT (10 μM) and RANKL (50 ng/ml). After 5–6 days of continuous intervention, proteins were extracted. The cells were washed three times with PBS, and 200 μl of RIPA lysate converted Phosphatase and Protease inhibitor was added to each well for 30 min on ice. Protein collection was performed using a cell scraper, and centrifugation was performed at 12,000 g, 5 min. The protein concentration was measured using a BCA kit (Biosharp, An Hui, China). The proteins were subjected to sodium dodecyl sulfate-polyacrylamide gel electrophoresis (SDS-PAGE) under constant pressure conditions of 80 V, 30 min, 120 V, and 70 min. The proteins were transferred onto the PVDF membrane at a constant current of 300 mA for 90 min. The membrane was then incubated with 5% bovine serum albumin (BSA) for 1 h, washed three times with TBS with Tween-20 (TSBT) for 10 min, incubated with the primary antibody overnight at 4°C, followed by the secondary antibody for 1 h at ambient temperature. Enhanced chemiluminescence (ECL) was used for visualization. The expression of related proteins was detected using β-actin as the internal control. Protein gray levels were quantified using ImageJ software (v. 1.48).

#### 2.2.8 Immunofluorescence staining of podosome belts and vinculin

RAW264.7 cells were cultivated in a 96-well cell culture apparatus at a density of 5 × 10^3^ cells and stimulated with 50 ng/ml RANKL (with or without DHT) for 6–7 days until the formation of mature osteoclasts. The cells were then fixed with 4% paraformaldehyde and blocked with 3% BSA in PBS for approximately 30 min.

200 μl of phalloidin diluent with the vinculin antibody was added to each well according to the Fibrous Actin kit procedure and incubated for 90 min in the dark. The cells were then incubated with the corresponding fluorescent anti-rabbit secondary antibody for 90 min at room temperature and observed using a fluorescence microscope.

## 3 Statistical analysis

All data are presented as mean ± standard deviation. Two independent samples were compared using the *t*-test. One-dimensional comparisons of the groups of samples were calculated using one-way ANOVA. *p <* 0.05 was considered significant.

## 4 Result

### 4.1 The common targets between dehydromiltirone and osteoporosis

**FIGURE 1:**
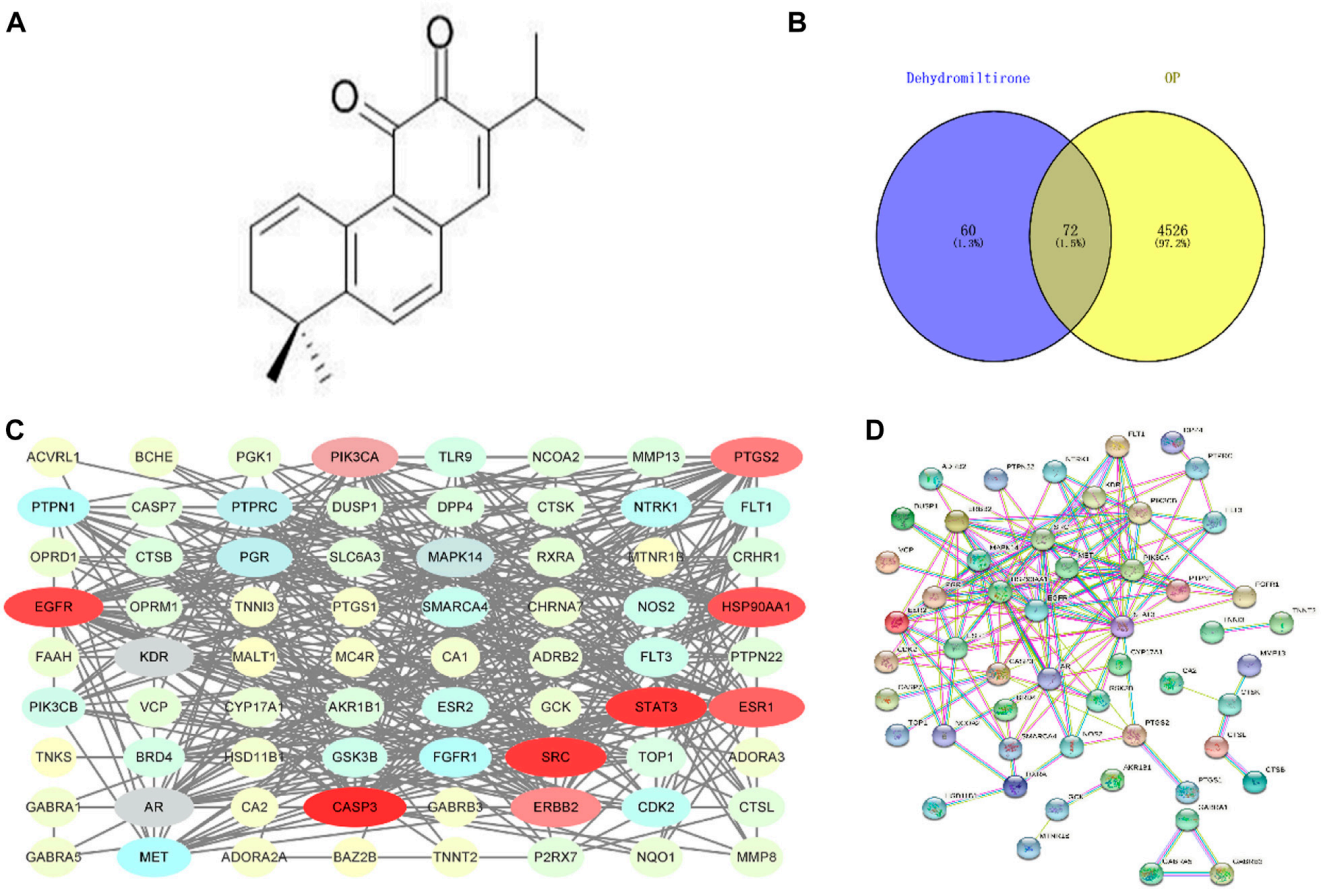
Molecular formula of dihydrotanshinone **(A)**; Venn diagram of the DHT-OP intersection targets **(B,C)**; and PPI network of the potential targets **(D)**.

The molecular formula of DHT is shown in Figure 1A. After searching and sorting, 4,598 OP-related disease targets were identified. The intersection of the target mapping was acquired, and 72 common targets were obtained, as shown in Figures 1B,C.

### 4.2 Construction of the protein-protein interaction network

The PPI results were obtained using the String database (HTTP://string-db.org), and the protein network was modularized using Cytoscape and the MCODE plug-in to obtain core proteins with a higher degree. The drug-disease protein interaction (PPI) network is shown in Figure 1D.

### 4.3 Biological function

**FIGURE 2:**
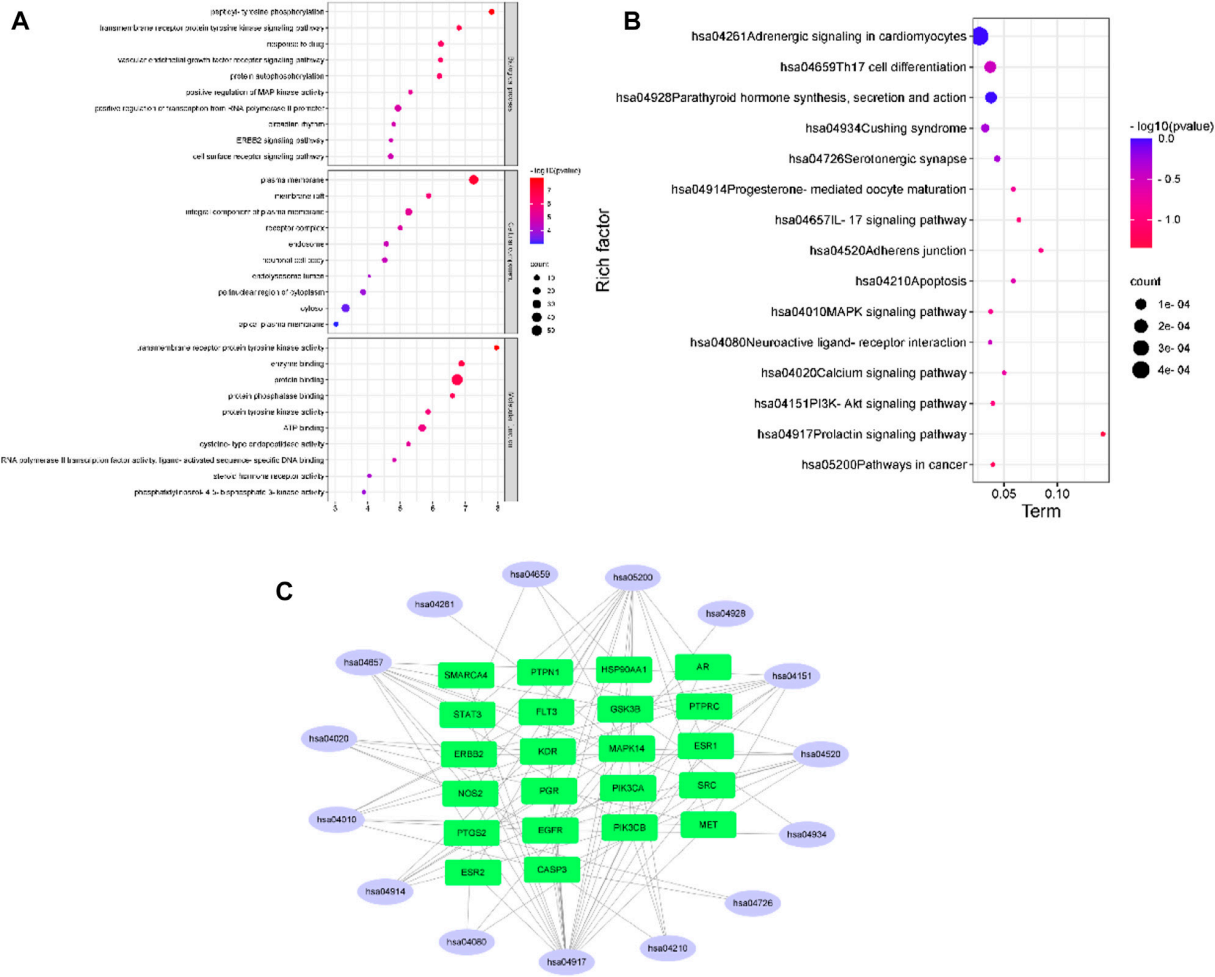
**(A,B)** GO and KEGG enrichment analysis **(C)** and pathway-target network.

A total of 213 results were obtained in the GO enrichment analysis, 127 of which were related to biological processes (BPs), including peptidyl-tyrosine phosphorylation, transmembrane receptor protein tyrosine kinase signaling pathway, and drug response; 30 related to cellular components (CCs), including the plasma membrane, membrane raft and integral component of the plasma membrane; and 56 related to molecular functions (MFs), including transmembrane receptor protein tyrosine kinase activity, enzyme binding, and protein binding, as shown in Figure 2A. KEGG enrichment analysis revealed a total of 143 signaling pathways, with cancer, PI3K-Akt, calcium, and MAPK signaling pathways as the most enriched pathways, as shown in Figure 2B. Figure 2C shows the distribution of the core targets in the KEGG signaling pathway. The results of GO and KEGG enrichment analyses suggest that DHT may play a joint regulatory role through the MAPK pathways, acting on certain proteins to suppress their phosphorylation.

### 4.4 Molecular docking

**FIGURE 3:**
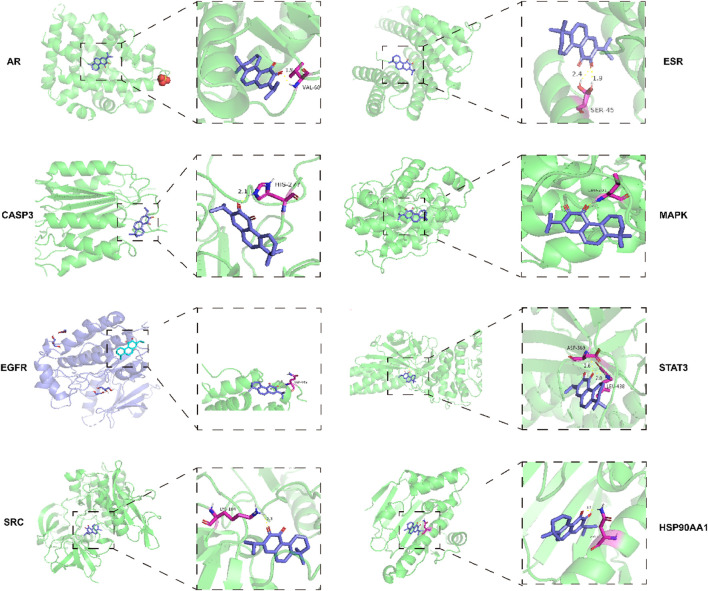
DHT docking with the core target molecule.

Table 2 lists the binding energies of ligands and receptors. All binding energies were less than −5 kcal/mol. The molecular docking results indicated that AR, SRC, MAPK14, CASP3, EGFR, STAT3, ESR1, HSP90AA1, and DHT were stable after docking, as shown in Figure 3.

**TABLE 2:** Molecular interactions between the core targets and DHT.

Compound	Target	Affinity (kcal/mol)
DHT	AR	−9.26
DHT	SRC	−8.24
DHT	MAPK14	−7.69
DHT	CASP3	−6.45
DHT	EGFR	−6.81
DHT	STAT3	−6.79
DHT	ESR1	−6.49
DHT	HSP90AA1	−7.83

### 4.5 CCK-8 assay

**FIGURE 4:**
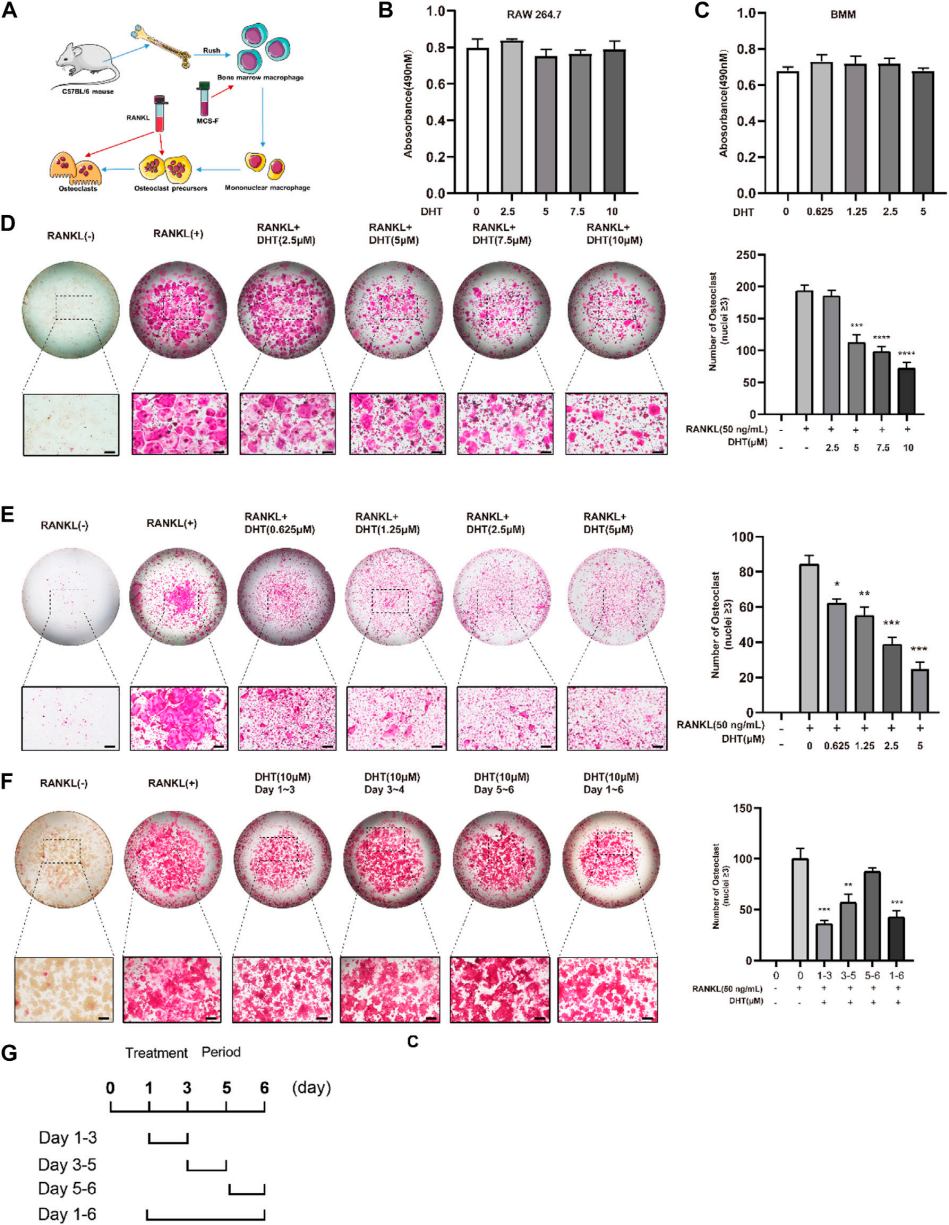
Dehydromiltirone (DHT) suppresses RANKL-induced osteoclastogenesis *in vitro*. **(A)** Schematic of osteoclast extraction, culture, and differentiation induction. **(B,C)** RAW264.7 and BMMs treated with different DHT concentrations were detected by the CCK-8 method. **(D,E)** Representative images of RAW264.7 cells and BMMs treated with various concentrations of DHT and the TRACP strains were obtained by light microscopy. **(F,G)** Stimulation with 10 μM at different stages of osteoclast differentiation for 1–3, 3–4, 5–6, and 1–6 days and the statistical analysis. *p < 0.05, **p < 0.01, ***p < 0.001, ****p < 0.0001 relative to the control group. Scale Bar = 200 μm.

A schematic of BMM extraction and culture was created for reference (Figure 4A). The cytotoxicity/proliferation of RAW264.7 and BMMs was detected using the CCK-8 assay, and cells were stimulated with various concentrations of DHT for 48 h. DHT had no cytotoxic or proliferative effects on RAW264.7 and BMMs at concentrations below 10 and 7.5 μM, respectively, compared to the controls (Figures 4B,C).

### 4.6 Dehydromiltirone inhibits RANKL-induced osteoclastogenesis

To investigate the effect of DHT on RANKL-induced osteoclast formation, RAW264.7 was simultaneously stimulated with RANKL and DHT, and BMMs were differentiated into osteoclasts containing RANKL (50 ng/ml) and M-CSF (50 ng/ml). DHT inhibited osteoclast formation in a concentration-dependent manner. For RAW264.7 and BMMs, the number of osteoclasts induced by DHT (10 μM for RAW264.7, 7.5 μM for BMMs) was significantly decreased relative to that induced by the positive control (Figures 4D,E).

RAW264.7 cells were treated (1–3, 3–5, 5–6 days) with DHT to determine the longer-lasting inhibitory effect of 10 µM DHT on osteoclasts. DHT had different effects on osteoclast differentiation at different time points and a significant effect at the double stage (day 1–3, 3–5, *p <* 0.05) (Figures 4F,G).

**FIGURE 5:**
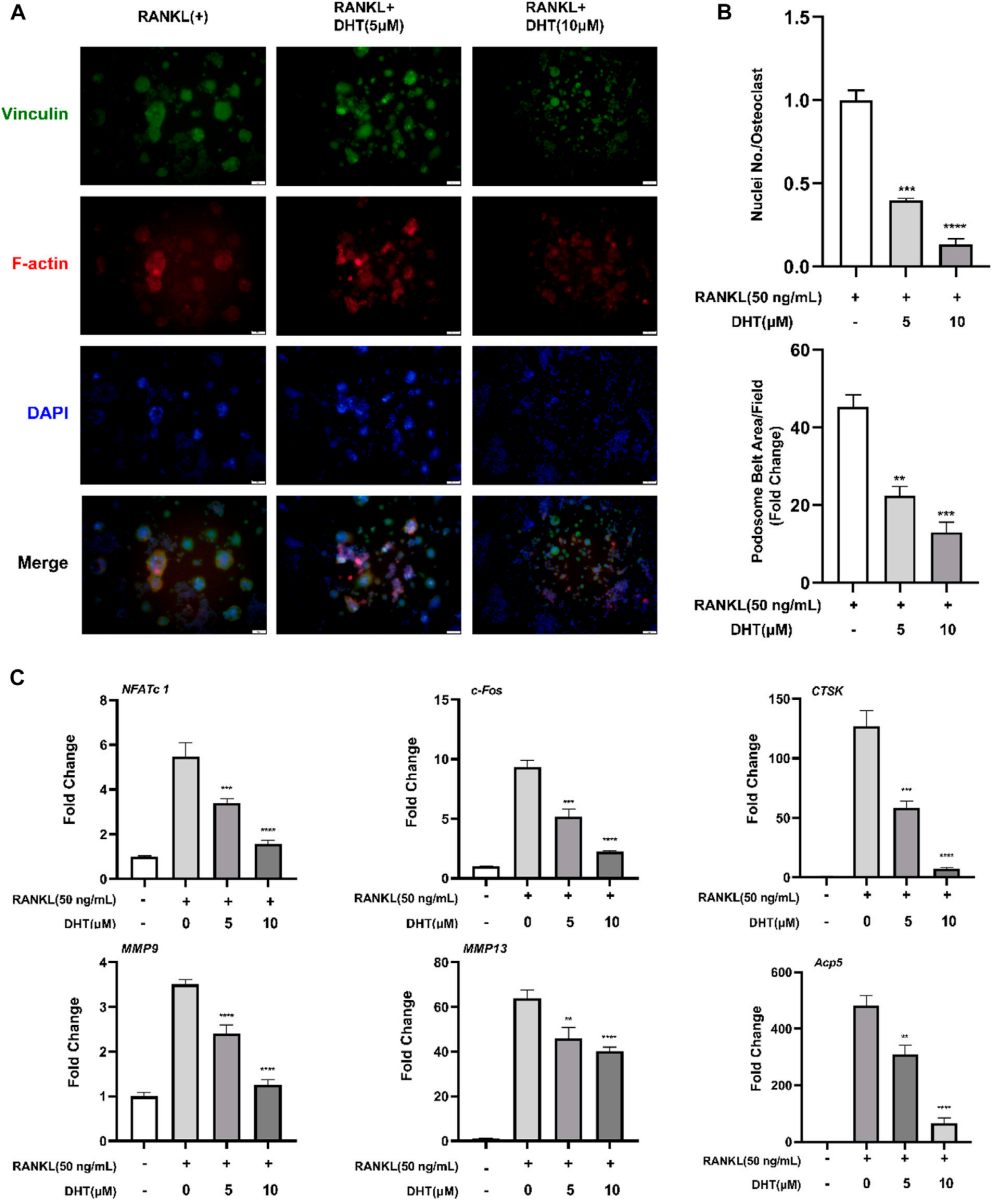
DHT inhibits the formation of podosome belts of osteoclasts induced by RANKL. **(A)** The Podosome Ring and nuclei were stained with Rhodamine Phalloidin, Vinculin, and DAPI, respectively, and confocal images were obtained. **(B)** The number of nuclei per osteoclast and the mean area of erythrocyte ligament per cell was quantified. **(C)**The expression of genes related to osteoclasts based on PCR, including *NFATc1, c-Fos*, *CTSK, MMP9, MMP13,* and *Acp5* (TRAcP). β-Actin expression was used to normalize the gene expression levels (n = 3). **p < 0.01, ***p < 0.001, ****p < 0.0001 relative to the RANKL-induced control group. Scale Bar = 200 μm.

The effect of DHT on the morphology and nuclear transport of osteoclasts was verified by staining Podosome belts and Vinculin. The formation of the F-actin podosome ring on the surface of the osteoclasts and aggregation of the nucleus was significantly inhibited by 5 and 10 μM DHT (Figures 5A,B).

### 4.7 Dehydromiltirone attenuates osteoclast‐involved gene expression

To further explore the mechanism of DHT inhibiting osteoclast formation, RAW264.7 cells were cultured in RANKL and treated with DHT (5 and 10 μM) for approximately 5–6 days until mature osteoclasts formed. RT-PCR detection of *NFATc1, c-Fos, CTSK, MMP9, MMP13*, and *Acp5* expression levels. As illustrated in Figure 5C, DHT effectively inhibited the expression of related genes in a dose-dependent concentration compared with the control group.

### 4.8 Dehydromiltirone inhibits osteoclastic resorption activity

**FIGURE 6:**
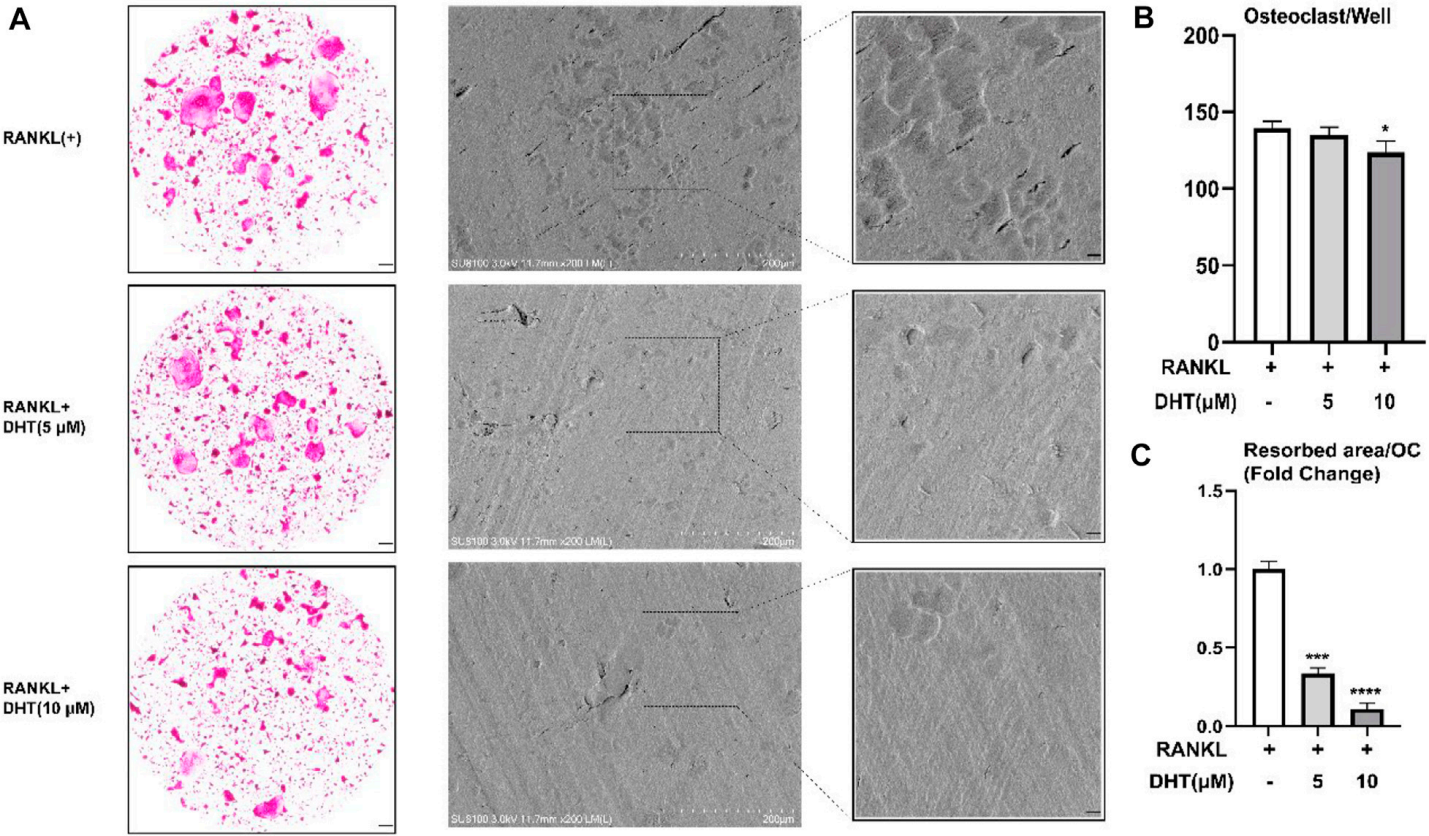
Effect of DHT on bone resorption of osteoclasts. **(A)** DHT interfered with mature osteoclasts for 48 h and found a statistical difference at 10 μM concentrations. **(B,C)**There was a significant difference between the number of osteoclasts and the area of bone resorption. *p < 0.05, ***p < 0.001, ****p < 0.0001 relative to the RANKL-induced control group. Scale Bar = 200 μm.

Next, we investigated the effect of DHT on the bone resorption of osteoclasts. DHT (5 μM, 10 μM) stimulated mature osteoclasts for 48 h, the bone resorption area, and the number of osteoclasts per well as shown in Figure 6. We observed that at a concentration of 10 μM, the number of osteoclasts still had a statistical difference compared with the control group. Besides, the area of bone resorption was significantly reduced, showing the characteristics of a concentration gradient.

### 4.9 Dehydromiltirone inhibits the expression of NFATc1 and related proteins

**FIGURE 7:**
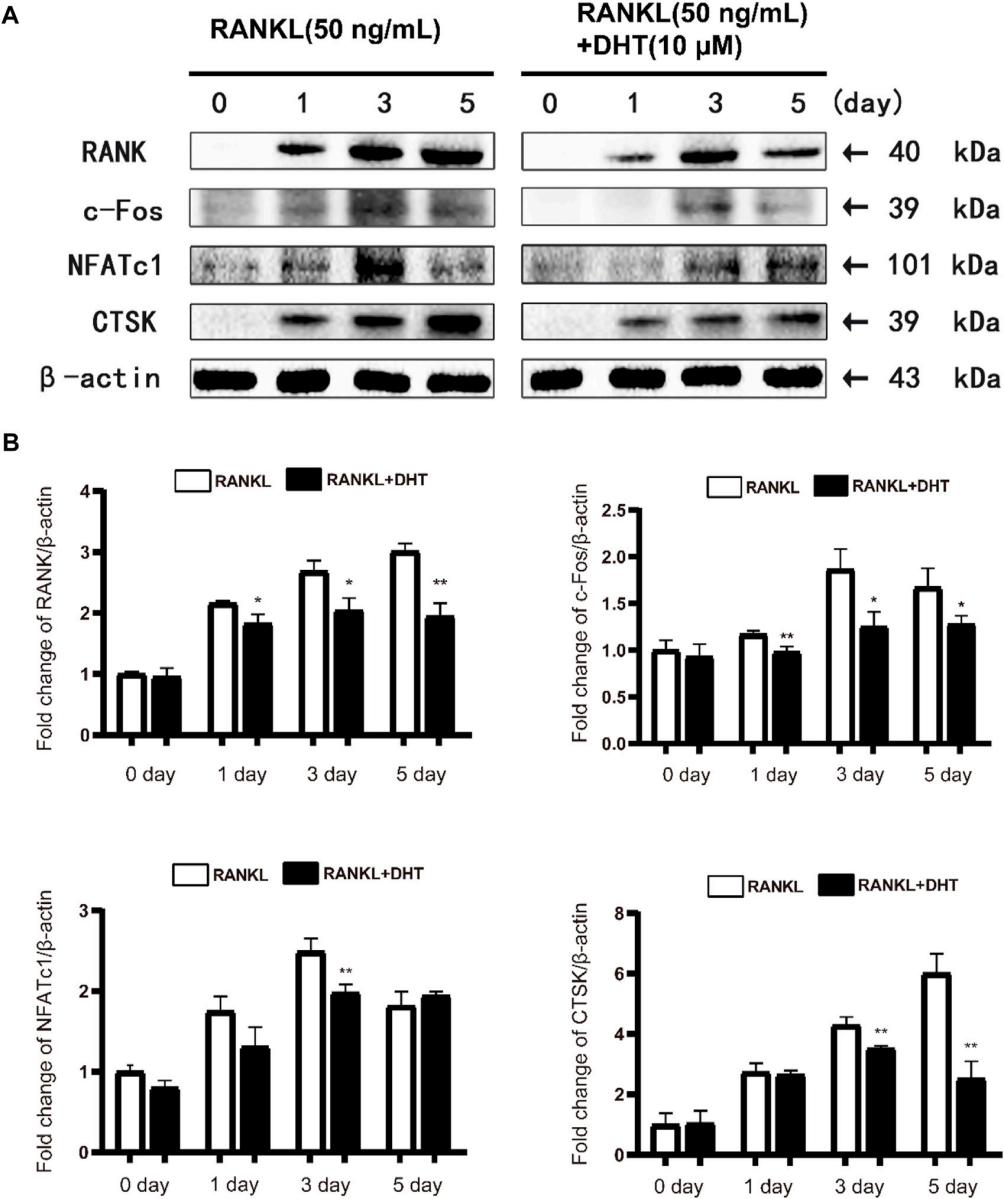
DHT can effectively inhibit the expression of osteoclast differentiation-related proteins**(A)**Representative images of the effect of DHT on RANKL stimulation (50 ng/ml) on days 0, 1, 3, and 5 for RANK, c-Fos, NFATc1, CTSK, and Acp5 protein. **(B)** Quantification of the ratio of band intensities to that of β-actin for RANK, c-Fos, NFATc1, and CTSK (*n* = 3). *p < 0.05, **p < 0.01 compared to the RANKL-induced group.

Western blot revealed that DHT significantly inhibited the expression of the RANK, c-Fos, NFATc1, and CTSK proteins declined following treatment with DHT compared to treatment with the positive control (Figure 7). Therefore, DHT may affect downstream signaling by inhibiting NFATc1 activity.

### 4.10 Dehydromiltirone represses the RANKL‐induced MAPK signaling pathway

**FIGURE 8:**
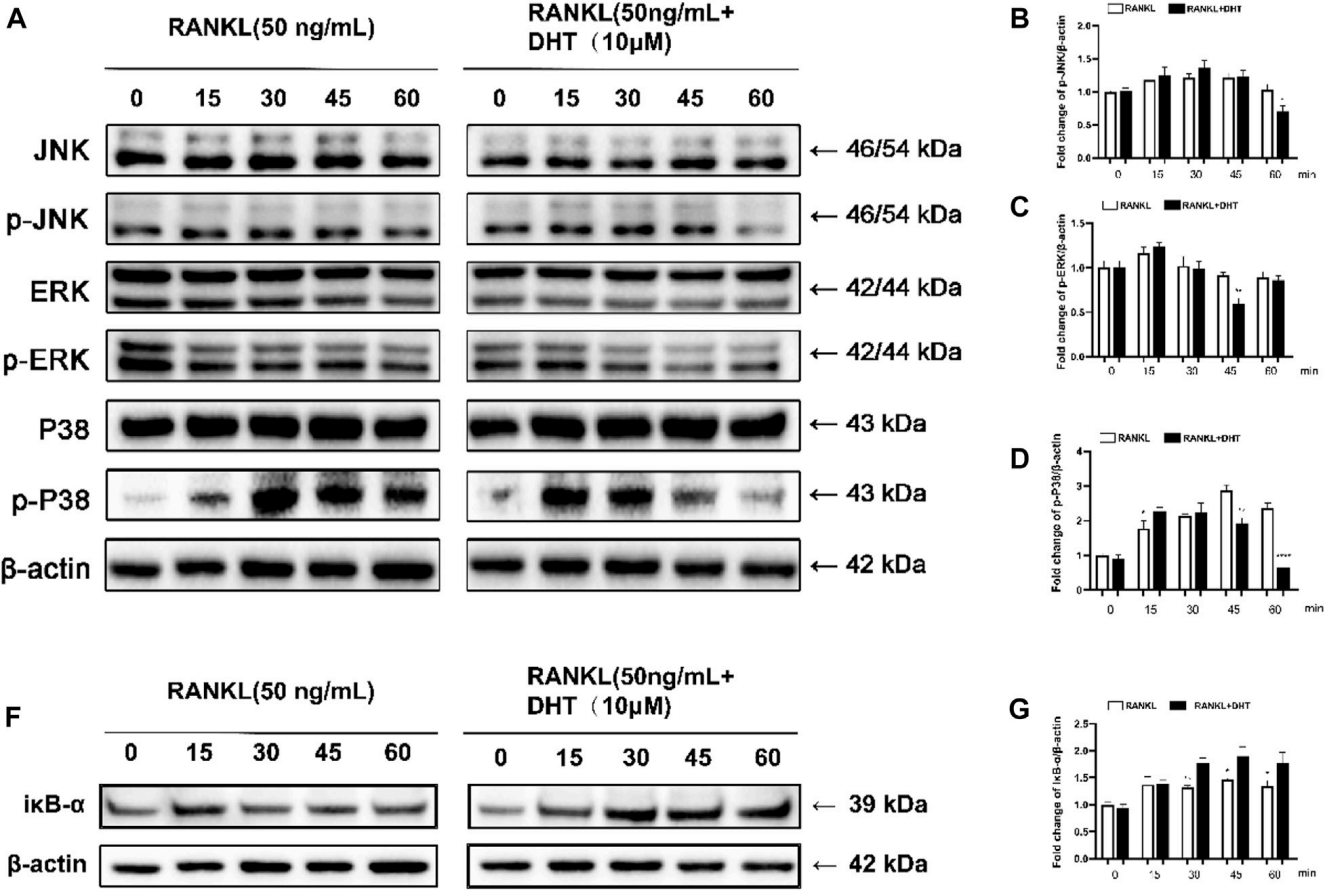
DHT inhibits RANKL-induced ERK activation and P38 phosphorylation. **(A)** p-JNK1/2, JNK, p-ERK1/2, ERK, p-P38, P38, and RANKL were stimulated with DHT dots (with or without 10 μM) at 0, 15, 30, 45, and 60 min **(B–D)** Quantification of the refractive index for the p-ERK, p-P38, and p-JNK bands. **(F,G)** The relative protein level of IκB‐α is standardized to that of β‐actin. (*n* = 3). *p < 0.05, **p < 0.01, ***p < 0.001, ****p < 0.0001 compared to the control group.

To further investigate the mechanism by which DHT inhibits osteoclast formation, we analyzed MAPK pathway-related proteins, including JNK, ERK, and P38, by western blotting. The phosphorylation levels of JNK, ERK, and P38 were measured at 0, 15, 30, 45, and 60 min after DHT stimulation. Based on previous literature, DHT can inhibit the expression of related inflammatory factors through the NF‐κB pathway (Guo et al., 2014; Qin et al., 2021), and NF‐κB is one of the classical signaling pathways of osteoclasts. Therefore, the pathway of NF‐κB was employed as the research object to explore the molecular mechanism by which DHT inhibits osteoclasts to exhibit an anti-osteoporosis effect. As illustrated in Figure 8, DHT significantly inhibited ERK1/2 and P38. After 45 min of RANKL stimulation, DHT significantly decreased the expression of phosphorylated p-ERK1/2 in RAW264.7 cells. DHT significantly inhibited p-JNK expression after 60 min and p38 phosphorylation at 45 min after RANKL stimulation. After treatment with DHT (10 μM) for 1 h, as illustrated, the Western blot revealed significant inhibition of IκB-α degradation by DHT, especially at 30 and 60 min. The above results suggest that DHT may inhibit MAPK/NF‐κB-induced signaling, including the phosphorylation of P38, ERK1/2, JNK, and the inhibition of IκB-α.

## 5 Discussion

Excessive bone resorption due to active osteoclasts is associated with but not limited to several bone diseases, including osteoporosis, rheumatoid arthritis, synovitis, periodontitis, cholesteatoma, and others (Lee et al., 2015; Boudin and Van Hul, 2017; Jamsen et al., 2017; Muramatsu et al., 2021). Osteoclasts are tissue-specific hematopoietic giant cells formed by the aggregation of several monocytes and macrophage progenitors on or near the bone surface. Mature osteoclasts secrete proteolytic and acid enzymes, such as Acp5, CTSK, and matrix metalloproteinases (MMPs), which are linked to the bone matrix and collagen formed by osteoblasts during bone resorption. Under pathological conditions, osteoporosis is often caused by hyperactivity of bone resorption, which affects normal physiological bone remodeling and excessive bone loss (Kim et al., 2020).

Osteoporosis, a high-prevalence clinical disease, is a public health burden on society, with approximately 10 million people diagnosed each year in the United States. Women in their 50 s and approximately one in five men are at increased risk of osteoporotic fractures (Weycker et al., 2016; Borgstrom et al., 2020; Ayub et al., 2021). Many drugs are used to treat osteoporosis, such as bisphosphonates, calcitonin, raloxifene, and denosumab (Anthamatten and Parish, 2019; Ensrud and Crandall, 2021; Kobayakawa et al., 2021). However, due to adverse reactions, such as bone end sclerosis (Ensrud and Crandall, 2021), multiple fractures after drug withdrawal (Deeks, 2018), osteonecrosis (Reid, 2015), and hypercalcemia (Roux et al., 2019), which ultimately lead to a limited range of drug applications, new drugs that are effective and have few side effects are required.

In addition to the development of modern information technology and further bioinformatics and pharmacology studies, researchers have combined network pharmacology and traditional Chinese medicine by analyzing the active components of traditional Chinese medicine (TCM) and constructing the “Compound-protein/gene-disease” interaction to explain the related biological function and mechanism of action between drug and disease (Wu et al., 2016; Zhang et al., 2019; Jiao et al., 2021). Thus, determining the pharmacological action and mechanism of TCM is of great significance to modern research and the development of new TCM drugs and their clinical application.

Our study revealed 72 targets between DHT and osteoporosis, including CTSK, MMP13, MAPK14, CASP3, etc., suggesting that these targets are principally related to the inflammatory response, apoptosis, and oxidative stress, which is consistent with the results of GO and KEGG analyses.

DHT could significantly reduce the expression of the CTSK gene and protein, which was consistent with the experimental results *in vitro*. CTSK is a cathepsin protein that is mainly expressed in osteoclasts and is involved in bone resorption and bone formation (Inaoka et al., 1995; Bromme and Lecaille, 2009). When CTSK is knocked out in mice, osteoclast bone resorption is reduced, thereby increasing bone formation by affecting the RANKL/OPG signaling pathway of osteoblasts, however, its knockdown may also contribute to the development of osteosclerosis (Lotinun et al., 2013). MMP13 belongs to the MMP family. The MMP family is mainly secreted by osteoblasts and selectively secreted by osteoclasts. (Freije et al., 1994; Ohshiba et al., 2003). In the present study, DHT decreased the expression of MMP13 and MMP9 or other genes involved in inflammation, which is consistent with the results reported in previous literature.

When RANKL binds to RANK, several sequential signaling cascades are initiated to govern the formation of mature osteoclasts. The MAPK pathways, including P38, ERK, and JNK, are involved in osteoclast differentiation and apoptosis (Mizukami et al., 2002; Wada et al., 2017). P38 plays a concerning role in the idiophone of osteoclast precursors into mature osteoclasts. When P38 is activated through the RANKL-RANK-TRAF6 axis, TRAF6 accumulates in the cytoplasmic tail, thereby promoting the differentiation of osteoclast progenitors into mature osteoclasts (Lee et al., 2016). Similarly, JNK and ERK play important roles in osteoclast apoptosis and precursor proliferation, respectively. ERK activation leads to an increase in AP-1 activity through c-Fos induction, which leads to an increase in c-Fos synthesis. The AP-1 protein structures formed by the binding of c-Fos to pre-existing Jun proteins transcribed in the nucleus are more stable than those formed by JUN alone (Karin, 1995). Consistent with the above conclusions, DHT inhibits JNK phosphorylation at 60 min and ERK and P38 phosphorylation at 45 and 60 min, respectively. Therefore, DHT may inhibit osteoclast formation by blocking the MAPK pathway.

NF-κB, a vital transcription factor in bone remodeling and inflammation, plays an important role in the regulation of osteoclast differentiation. The NF-κB complex binds to the IκB-α protein to prevent nuclear translocation. However, after RANKL stimulation, NF-κB is degraded and released into the cytoplasm, inducing the generation of mature osteoclasts (Lawrence, 2009; Yao et al., 2021). Based on our results, DHT can inhibit the degradation of IκB-α upon RANKL stimulation, suggesting that it inhibits the activity of NF-κB.

**FIGURE 9:**
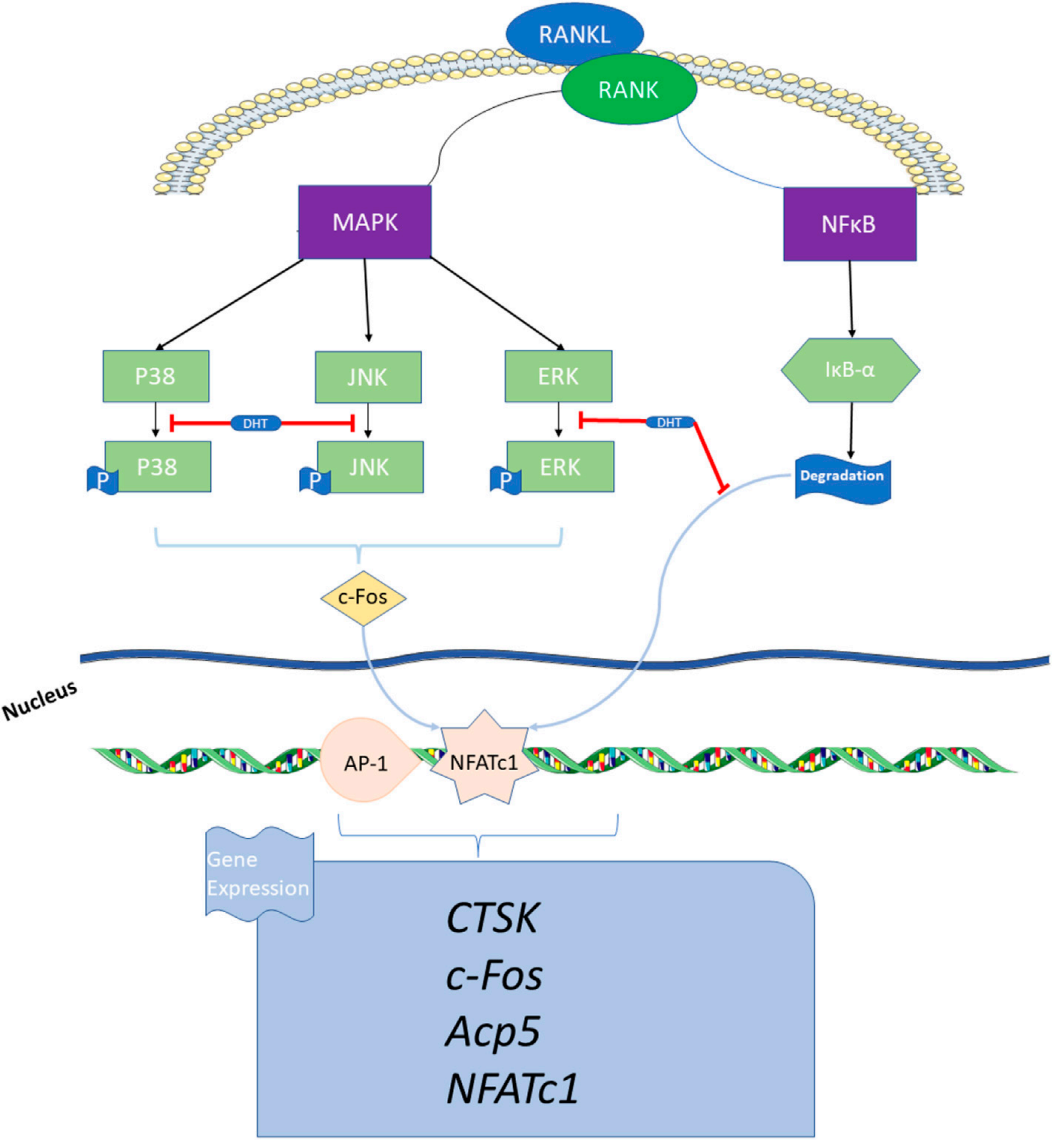
Schematic indicating the mechanism by which DHT inhibits osteoclast formation. DHT inhibits RANKL-induced osteoclast differentiation *via* the MAPK and NF-κB signaling pathways, thereby decreasing the expression of related genes and proteins, such as NFATc1, Acp5, c-Fos, and CTSK that affect osteoclast bone resorption.

In conclusion, DHT is not cytotoxic to RAW264.7 and BMMs, and exhibits anti-osteoporotic functions at working concentrations of 10 and 7.5 μM, respectively. DHT was found to inhibit RANKL-induced osteoclast preparations and bone resorption by affecting the MAPK and NF-κB pathways, aligning with the network pharmacology results. Based on such findings, researchers have shown strong support for the following points:1) DHT can promote the development of traditional Chinese medicine by adding modern bio-information technology into the research system of traditional Chinese medicine; 2): Theory and preliminary experiments support that DHT, a natural compound of *Salvia miltiorrhiza Bge* and *Salvia przewalskii Maxim*, can affect osteoclast differentiation at lower concentrations, and potentially inhibiting osteoporosis caused by excessive-resorption through inhibiting osteoclast formation. However, the specific mechanism of action of DHT in the inhibition of osteoclast production and anti-osteoporosis still needs further validation *via in vivo* and related experiments, which is the direction of future studies (Figure 9).

## Data Availability

The datasets presented in this study can be found in online repositories. The names of the repository/repositories and accession number(s) can be found in the article/supplementary material.
